# Beyond “somatization” and “psychologization”: symptom-level variation in depressed Han Chinese and Euro-Canadian outpatients

**DOI:** 10.3389/fpsyg.2013.00377

**Published:** 2013-06-27

**Authors:** Jessica Dere, Jiahong Sun, Yue Zhao, Tonje J. Persson, Xiongzhao Zhu, Shuqiao Yao, R. Michael Bagby, Andrew G. Ryder

**Affiliations:** ^1^Social Aetiology of Mental Illness CIHR Training Program, Centre for Addiction and Mental Health, University of TorontoToronto, ON, Canada; ^2^Department of Psychology, Concordia UniversityMontreal, QC, Canada; ^3^Medical Psychological Institute of the Second Xiangya Hospital, Central South UniversityChangsha, China; ^4^Departments of Psychology and Psychiatry, University of TorontoToronto, ON, Canada; ^5^Campbell Family Mental Health Research Institute, Centre for Addiction and Mental HealthToronto, ON, Canada; ^6^Lady Davis Institute and the Culture and Mental Health Research Unit, SMBD–Jewish General HospitalMontreal, QC, Canada

**Keywords:** depression, Chinese, Euro-Canadian, symptom presentation, differential item functioning, cultural-clinical psychology

## Abstract

The finding that people of Chinese heritage tend to emphasize somatic rather than psychological symptoms of depression has frequently been discussed in the culture and mental health literature since the 1970s. Recent studies have confirmed that Chinese samples report more somatic and fewer psychological depression symptoms compared to “Western” samples. The question remains, however, as to whether or not these effects are attributable to variation in all the constituent symptoms or to a subset. If the latter, there is the additional possibility that some symptoms might show a divergent pattern. Such findings would have implications for how cultural variations in symptom presentation are interpreted, and would also inform the cultural study of affective experiences more broadly. The current study addressed these issues in Chinese (*n* = 175) and Euro-Canadian (*n* = 107) psychiatric outpatients originally described by Ryder et al. (2008). Differential item functioning (DIF) was used to examine whether specific somatic and psychological symptoms diverged from the overall patterns of cultural variation. Chi-square analyses were used to examine atypical somatic symptoms (e.g., hypersomnia), previously neglected in this literature. No DIF was observed for the typical somatic symptoms, but Euro-Canadians reported greater levels of atypical somatic symptoms, and showed higher rates of atypical depression. DIF was observed for psychological symptoms—the Chinese reported high levels of “suppressed emotions” and “depressed mood,” relative to their overall psychological symptom reporting. Chinese outpatients also spontaneously reported “depressed mood” at similar levels as the Euro-Canadians, contrary to prevailing ideas about Chinese unwillingness to discuss depression. Overall, the findings provide a more nuanced picture of how culture shapes symptom presentation and point toward future studies designed to unpack cultural variation in narrower subsets of depressive symptoms.

The finding that people of Chinese heritage tend to emphasize somatic rather than psychological symptoms of depression has frequently been discussed in the interdisciplinary literature on culture and mental health. Indeed, this phenomenon is among the most well-known examples of cultural variation in psychopathology, central to the shift toward “the new cross-cultural psychiatry” in the late 1970s and 1980s (Kleinman, 1977, 1982) and also as a spur to developments in cultural-clinical psychology (Ryder et al., 2011; Ryder and Chentsova-Dutton, 2012). Many explanations have been proposed and, in recent years, cross-national evidence for Chinese somatization—and “Western psychologization”—has been established (Parker et al., 2001a; Ryder et al., 2008). Nonetheless, our understanding of this phenomenon remains limited.

One issue is that most research in this area has focused on broad symptom sets, rather than individual symptoms. While symptom sets facilitate the development of psychometrically robust measurement instruments, there is a tendency to interpret the results as pertaining to *all* somatic and psychological symptoms. Recent research suggests that such generalization may be premature. In a follow-up study using Ryder and colleagues' (2008) data, Zhou and colleagues (2011) confirmed that Chinese outpatients had a higher self-reported tendency to focus on somatic symptoms when depressed compared to Euro-Canadian outpatients. At the same time, however, these same Chinese outpatients had a *lower* self-reported tendency to focus on somatic symptoms when anxious. Claims that Chinese patients focus on the body in general, or deny emotions in general, miss out on the more nuanced picture that is emerging from the literature.

The work of Zhou and colleagues (2011) compares depression to anxiety; what would happen were similar qualifications and exceptions to be found within the domain of depressive symptoms? One important distinction between symptom scales and other psychometric instruments is that the individual items convey clinically important information. For example, items constituting an interdependent self-construal scale are important to the extent that they contribute to a total score that validly measures interdependent self-construal; whether a particular respondent endorses a particular item is rarely considered. For symptom measures, in contrast, each item is important as a specific representation of a respondent's experience. Our understanding of somatization or psychologization may be changed markedly if we find that certain symptoms do not follow the expected cultural pattern, especially if that symptom is central to our understanding of what depression is – we would describe the phenomenon differently, assess it differently, perhaps even explain and treat it differently. The current study aims to move in precisely this direction.

## “Chinese somatization”

Chinese somatization has primarily attracted attention due to a contrast with the supposed “Western” emphasis on psychological symptoms. Kleinman (1982) inaugurated this line of inquiry in his original field study, which employed anthropological and psychiatric research methods in examining 100 Chinese patients diagnosed with neurasthenia. This diagnostic category overlaps with depression but emphasizes somatic symptoms, including fatigue, sleep problems, and muscle pain; the diagnosis was commonly used in China at the time (Ryder and Chentsova-Dutton, 2012). The study's most remarkable finding was that although somatic complaints were central to the patients' reported distress, 87 out of the 100 patients could be re-diagnosed as having some form of depression based on criteria in the *Diagnostic and Statistical Manual of Mental Disorders* (3rd Edn.; *DSM-III*; American Psychiatric Association, 1980). Kleinman's work became sufficiently well-known that his findings have been invoked to help explain the remarkably low rates of depression reported in China during this time (Parker et al., 2001b; Ryder and Chentsova-Dutton, 2012).

Before delving further into more recent empirical findings, the term “somatization” merits some critical examination. As noted by a number of scholars and mental health practitioners, the term “somatization” can refer to a number of different phenomena (e.g., Kirmayer and Robbins, 1991; Kirmayer and Young, 1998; Simon et al., 1999). To begin with, there is the confusion between somatization as somatoform disorder, somatization as an aspect of hypochondriasis, and somatization as a mode of symptom presentation (Kirmayer and Robbins, 1991). This last form of somatization—“presenting somatization”—is our focus here, and can be broken down further. First, it can be understood as a form of help-seeking behavior, whereby patients with psychological distress tend to emphasize somatic complaints when describing their symptoms. This tendency may be observed when somatic symptoms are perceived as providing more effective access to health care resources and/or when psychological symptoms are perceived as stigmatizing. Second, it can be understood as “somatosensory amplification,” emphasizing the association between depression and medically unexplained somatic symptoms. According to this view, bodily sensations are experienced as particularly intense and disturbing among certain psychiatric patients (Barsky, 1992), which can give rise to higher rates of somatic symptom reporting. Third, it can be understood as the exclusive reporting of somatic symptoms, accompanied by the denial of any psychological distress.

Simon and colleagues (1999) directly tested these three approaches to presenting somatization among 1146 patients across 14 countries on 5 continents, including China. They found that multiple unexplained somatic symptoms were common among all centers and the balance of psychological and somatic symptoms was similar across sites. These authors concluded that there were no systematic differences in levels of somatization based on the second and third definitions outlined above. In contrast, it was found that there was considerable variation in somatic symptom presentation across research sites when using the first definition of somatization, regarding the emphasis on somatic symptoms when seeking medical services. Specifically, patients attending walk-in centers with no ongoing relationship with a physician were more likely to report somatic symptoms as compared to those who were visiting their regular family physician. The authors concluded that cultural differences in “somatization” can essentially be understood in the context of symptom presentation, rather than as a denial or lack of psychological symptoms.

This idea that somatic symptom emphasis represents a culturally shaped pattern of symptom presentation rather than an inability to experience or describe psychological symptoms has been supported by cross-national clinical studies. Parker and colleagues (2001a) found that a greater percentage of Malaysian Chinese outpatients, as compared to Euro-Australian outpatients, nominated a somatic symptom as their primary presenting complaint, 60% vs. 13%. At the same time, however, both groups endorsed a number of both somatic and psychological symptoms on a self-report symptom inventory, with neither group showing an exclusively somatic nor psychological symptom profile. In the most recent cross-national clinical study to address this topic, Ryder and colleagues (2008) found that Chinese outpatients were significantly more likely to report somatic symptoms on unstructured and structured interviews than their Euro-Canadian counterparts; however, there was no difference in somatic symptom reporting on a self-report questionnaire. The group difference on the unstructured interview was markedly reduced in a follow-up analysis that controlled for demographics. In short, the only situation in which Chinese patients were clearly more likely to emphasize somatic symptoms was when an unfamiliar clinician interviewed them in a structured way, once again supporting the notion that aspects of the patient role can shape symptom reporting.

The inclusion of somatic symptoms in the *DSM* diagnostic criteria for major depression, along with empirical evidence from the depression and somatization literature, suggest that the presentation of somatic symptoms in the context of depression is by no means unique to any one cultural context. Chinese somatization is best seen as a matter of symptom *emphasis*, rather than a presentation pattern that excludes or denies psychological symptoms. However, it is worth noting at this point that the very concept of somatization rests on the cultural assumption that psychological symptoms are more central to depression than somatic symptoms. It is equally as legitimate to study the phenomenon of “Western psychologization” as it is to study “Chinese somatization” (Ryder and Chentsova-Dutton, 2012). Indeed, the two cross-national studies discussed above (Parker et al., 2001a; Ryder et al., 2008) report similar findings regarding a “Western” tendency to emphasize psychological symptoms of depression. The authors of both studies noted a stronger and more consistent cultural group difference in the reporting of psychological symptoms as compared with the reporting of somatic symptoms. This raises the possibility that differences in psychological symptom reporting may represent an even more pronounced cultural variation than differences in somatic symptom reporting, and suggests that “Western psychologization” warrants further study.

Our discussion so far suggests that somatic and psychological symptoms represent two symptom sets that can be endorsed to different degrees by respondents from different cultural groups. When examining these two broad symptom categories, however, the question arises as to the role that each individual symptom plays in driving overall cultural group differences in symptom presentation. As discussed earlier, this question is important given that each symptom is itself a clinically important aspect of a respondent's experience and is potentially relevant to how we understand the cultural variations under consideration. In contrast to earlier studies, which have only examined clinical presentation at the level of these broad symptom sets, the current analyses are specifically designed to investigate variation in the reporting of individual symptoms. We are interested in examining whether the symptoms that fall under these two broad symptom categories show consistent differences between Chinese and Euro-Canadian outpatients, or whether the observed cultural variations are better explained by a particular subset of symptoms.

Having provided a general overview of the Chinese somatization literature and introduced the focus of the current study, the remaining introductory sections will serve to further situate this work. We will first provide a brief commentary linking the current work with the emerging field of cultural-clinical psychology (Ryder et al., 2011; Ryder and Chentsova-Dutton, 2012). Next, we will consider some problems that may arise with the current method of only looking at broadly defined symptom categories. We will then review evidence found in previous studies that sparked our interest in studying cultural variation at the level of individual symptoms. Finally, since the study's central research question is tested using a statistical technique called differential item functioning, we will introduce this technique and provide a brief discussion of the rationale for its use.

## Somatization, psychologization, and cultural-clinical psychology

Our discussion of the somatization and depression literature to this point has been primarily concerned with the value of incorporating a cultural perspective into the study of clinical phenomena; we have not directly addressed the related question of what cultural researchers might gain from clinically-focused work such as the current study. As Ryder and colleagues (2011) have emphasized, “[r]esearch in cultural–clinical psychology should tell us something new about the cultural contexts under study, not just the pathologies” (p. 962). Keeping this requirement in mind, we will briefly discuss some ways in which the questions addressed in the current study, as well as the methodology employed, are relevant to cultural psychology researchers who are not necessarily concerned with clinical research *per se*.

Emotion researchers generally study only one or a small number of emotional states at a time, and the psychological and somatic aspects of affective experience are rarely examined together (e.g., see Dzokoto, 2010). Studying a syndrome such as depression, which is constituted by a number and variety of symptoms, forces us to examine a constellation of mental and bodily states at once. Such work can thereby offer important lessons to cultural researchers, by emphasizing the co-occurrence of multiple states and the ways in which these groupings can differ across cultural contexts. Furthermore, the study of different clinical syndromes can provide important insights into the ways in which presumed patterns of cultural variation may not hold across forms of distress (e.g., Zhou et al., 2011).

Clinical research can also inform the work of cultural researchers by helping to refine our understanding of “cultural scripts.” Cultural scripts have recently been discussed as a valuable conceptual tool in cultural-clinical psychology, as they serve to link together cultural meanings and practices (see Ryder et al., 2011; Ryder and Chentsova-Dutton, 2012; Ryder et al., 2012). These scripts refer to organized units of culturally salient knowledge, which can be automatically retrieved, and serve to guide behaviors while also shaping the meaning and interpretation of such behaviors in a given cultural context. In general terms, examination of the content and function of cultural scripts can be seen as a central objective in cultural psychology research. We believe that the cultural study of clinical processes offers a distinct opportunity to reveal elements and/or variants of cultural scripts that are not easily accessed otherwise (see Ryder et al., 2011).

Examination of the concerns and experiences that are emphasized at times of distress within a given cultural context provides a window into particularly salient cultural themes. Conducting analyses at the level of individual symptoms rather than symptom subscales may serve to provide a more fine-grained picture of such themes. For example, the method employed in the current study will allow us to examine whether Chinese outpatients consistently report lower levels of psychological symptoms compared to Euro-Canadians, or if there are a subset of such symptoms that show a divergent pattern. Either result will carry implications for cultural researchers who study emotion, by speaking to the extent to which the negative psychological experiences implicated in depression can be understood as a tightly knit set. By examining those specific symptoms that show particularly strong cultural differences, our results can help to refine our understanding of cultural scripts in both Chinese and Euro-Canadian cultural contexts.

Finally, the study of clinical phenomena also offers novel constructs that can inform the work of cultural researchers. For example, the distinction between typical and atypical somatic symptoms—to be elaborated below—is rooted in clinical depression, but may well prove important for researchers studying cultural variations in somatic awareness and the link between somatic and emotional states (e.g., Chen et al., 2003; Tsai et al., 2004; Dzokoto, 2010). A more nuanced examination of somatization, such as in the current study, may reveal novel findings that challenge and refine the general notion that East Asian cultural contexts promote greater attention to somatic experiences as compared to “Western” cultural contexts.

## Problems with symptom categories

The *DSM-IV* (American Psychiatric Association, 1994) diagnostic criteria for major depression contain a number of somatic symptoms, some of which have a diametrically opposed “twin” symptom (e.g., weight loss vs. weight gain). In most of the depression studies we reviewed, symptom factors or subscales were calculated for the purposes of group comparison. Somatic subscales are almost always comprised of typical somatic symptoms, which include weight loss, decreased appetite, insomnia, psychomotor problems, and fatigue, with some variations in the specific item content across studies. “Atypical” somatic symptoms—including hypersomnia and hyperphagia (i.e., weight gain and/or increased appetite)—are usually excluded from somatic factors for conceptual as well as statistical reasons. Just as it is impossible for weight gain and weight loss to be simultaneously endorsed as symptoms, the inclusion of this type of item pair in a single subscale is statistically problematic. We consequently know very little about the cross-national distribution of atypical somatic symptoms.

Moreover, the atypical depression (AD) subtype is rarely studied in “non-Western” cultural contexts, even though it is associated with higher rates of somatization in “Western” samples (Seemüller et al., 2008; Thuile et al., 2009). As defined by *DSM-IV*, this depression subtype mixes atypical somatic symptoms with mood reactivity, heaviness in head and/or limbs, and interpersonal rejection sensitivity. There is reasonable evidence to support the co-occurrence of hypersomnia, hyperphagia, and interpersonal rejection sensitivity, although the other two symptoms appear problematic (Angst et al., 2006; Parker et al., 2002). Clinical studies conducted among North American samples show that between 28.0 and 43.0% of those with unipolar or bipolar depression meet the AD criteria (Robertson et al., 1996; Benazzi, 1999; Akiskal and Benazzi, 2005), and the lifetime prevalence rates among community samples range from 0.7 to 4.0% (Horwath et al., 1992; Sullivan et al., 1998). In a Hong Kong community sample, Lee and colleagues (2009) reported that the 12 month prevalence of AD (1.3%) was similar to that found among “Western” samples. However, no study to date has specifically examined atypical symptoms or the AD subtype in Mainland China. In examining both typical and atypical somatic symptoms, the current study is well-positioned to explore the AD subtype and its associated somatic symptoms, in a cross-national clinical comparison that includes Chinese outpatients.

## Symptom-level analysis

Beyond the neglect of atypical depression, the focus on broadly defined symptom categories may have also resulted in a failure to recognize more nuanced cultural variations in depressive presentations. Kleinman (1982) hinted in his seminal study that even though the 87 Chinese patients diagnosed with neurasthenia could be re-diagnosed as having *DSM*-defined major depression, their symptom profile was strikingly different from what a prototypical “Western” depressed patient would display. Specifically, he pointed out that depressed mood was only endorsed by 9% of the Chinese patients, while their chief complaints were headaches (90%), insomnia (78%), dizziness (73%), and pain (48%). Therefore, it is important to bear in mind that depression can manifest itself in vastly different ways from one context to another, even when *DSM* diagnostic criteria are being met. Lack of attention to individual symptoms leads to an incomplete picture of this variation.

A closer inspection of Parker and colleagues' (2001a) findings also suggests that a simple group difference in somatic vs. psychological symptom reporting does not capture the full story. As mentioned above, these authors found that Malaysian Chinese outpatients were far more likely to report a somatic symptom as their most important presenting problem, whereas Euro-Australians were more likely to report a mood or cognitive symptom. However, examination of their findings suggests that the Malaysian Chinese group also reported a greater array of somatic symptoms as compared to the Euro-Australians; conversely, the list of cognitive symptoms nominated by the Euro-Australians was substantially longer than that of the Malaysian Chinese. Such symptom-level differences are not captured in analyses that rely on symptom subscales, and point toward the value of examining symptom presentation using various methods.

Parker and colleagues (2001a) also suggested the importance of examining cultural variation at the level of individual symptoms, and employed an item response theory (IRT) approach with the goal of determining whether their two groups differed in the severity threshold required to endorse particular symptoms. Using Rasch modeling, they examined how the probability of answering “yes” to individual symptoms related to participants' response level on the underlying dimension of interest (i.e., depression). They found considerable variation between their two groups with regards to the level of severity at which particular symptoms were more likely than not to be endorsed. For example, the Malaysian Chinese showed a lower threshold for endorsing the item “health problems” than their Euro-Australian counterparts, even though the actual prevalence of this item was similar for the two groups (76% and 66%, respectively). The Malaysian Chinese also showed a lower threshold for reporting “chest pain”; they were more likely to report this symptom at a lower level of depression severity as compared to the Euro-Australians. Examining differences in the threshold required for endorsing specific symptoms is one approach to building a more nuanced understanding of cultural variations in symptom reporting, above and beyond observed differences in symptom subscales.

## Differential item functioning

The discussion above makes the case for supplementing our study of broad somatic and psychological symptom subscales with the examination of individual symptoms or subsets of symptoms. The question to be answered is whether each symptom follows the overall pattern, or whether a particular subgroup of symptoms is primarily responsible for the observed effects. From a statistical perspective, techniques to examine differential item functioning (DIF) are ideally suited for this task. Broadly speaking, DIF occurs when groups vary with regards to the probability of answering a particular question in a certain way, holding constant group differences in the total score (Hambleton et al., 1991). DIF analysis provides an indication of unexpected behavior by an item on a scale, and statistically identifies items that function differently from one examinee group to another.

A variety of DIF detection methods exist, any of which can be applied to one of two purposes. The first purpose is to examine questions of measurement invariance across different groups, traditionally used in the context of test validation (Zumbo, 2007). Here, the goal is to determine whether there are any individual items that behave so differently across groups that they would compromise the total score to the extent that false conclusions might be drawn from group comparisons. Relatively high thresholds for identifying DIF are recommended for this task; small amounts of DIF, while potentially interesting, have a minimal impact on composite scales. This approach is a good fit for research questions where the underlying construct is the primary focus and individual items matter only to the extent that they are useful indicators of that construct.

The second purpose is to specifically search for DIF that might point to meaningful group variations worthy of further investigation. As DIF in this instance may be meaningful without necessarily compromising total scores, lower thresholds for identifying DIF are reasonable. This second purpose is the primary aim of the current study. Whereas Ryder and colleagues (2008) aimed to establish an acceptable level of measurement equivalence before proceeding to test levels of somatization and psychologization across groups, this study aims to determine how these terms ought to be defined. This approach is a good fit for situations where individual items have a primary importance beyond simply contributing to measurement of a more abstract construct. In the current study, we therefore return to Ryder and colleagues' (2008) data to ask: what symptom experiences precisely characterize the constructs of “somatization” and “psychologization” in Chinese and North American cultural contexts?

## Methods

### Sites

#### Changsha

The Chinese sample consisted of Han Chinese psychiatric outpatients, from the Neurosis Clinic of the Center for Psychological Research, at the Second Affiliated Hospital of Hunan Medical University, in Changsha, Hunan, People's Republic of China^1^. Participants were recruited in the Spring and Summer of 2002. China has a median age of 34.1 years and a life expectancy of 73.5 years (Central Intelligence Agency, 2009). The city of Changsha is the capital of Hunan province, located in south-central China; the metropolitan area had a population of approximately 6 million people in 2000 (National Bureau of Statistics of China, 2002).

The hospital where the data collection took place is one of ten key medical colleges co-founded by the Ministry of Education and Ministry of Health in China, and is one of the top 100 tertiary care hospitals in the country. The Neurosis Clinic is an outpatient psychiatric clinic, which was established in 1994. This clinic is the leading site in the region for the treatment of a range of psychiatric problems, including those that in North America would be termed mood, anxiety, somatoform, and personality disorders. Complex cases are often referred there and, by virtue of its medical school affiliation, the clinic has mandates for teaching and research as well as for clinical work. The patient base has increased significantly over the past decade; the clinic is open 6 days a week and receives approximately 25 patients a day, who usually self-refer to the clinic. Licensed psychiatrists provide the majority of clinical care, while psychology graduate students provide clinical administration services. It is worth noting that by having a catchment area that is predominantly a modernized urban center, comparisons with a North American setting are expected to yield smaller cultural differences than may be found in less urbanized Chinese sites.

Prior to their first visit to the Neurosis clinic, clients are assessed by a brief telephone interview in order to help determine their treatment eligibility. Those with severe psychiatric disorders are referred to the Inpatient Psychiatry Department of the hospital. Clinic appointments consist of a clinical interview, which is sometimes supplemented by psychological assessment tools, followed by the prescription of psychopharmacological and/or psychological interventions. Patients may return to the clinic for follow-up sessions based on clinical necessity.

With regards to the broader mental health system in which this site is situated, the vast majority of mental health professionals in China are psychiatrists or psychiatric nurses, with few clinical psychologists and social workers, and no occupational therapists. Psychiatrists and licensed psychiatric nurses are accredited by the Ministry of Health, psychological counselors by the Ministry of Human Resources and Social Security, and psychotherapists by both Ministries. According to the 2011 Mental Health Atlas from the World Health Organization (WHO, 2011), China has approximately 1.5 psychiatrists, 2.7 nurses, and 0.2 psychologists working in the mental health sector per 100,000 population. Contrast these numbers with Canada, where there are 12.6 psychiatrists, 65.0 nurses, and 46.6 psychologists in the mental health sector per 100,000 population. These numbers highlight the substantial personnel shortages facing China's mental health system, which currently represents one of the system's greatest challenges (Liu et al., 2011). These statistics suggest that patients in China who have received formalized mental health services represent a more select minority than those in Canada. However, these statistics cannot speak to the potential role of alternative types of practitioners and services, such as Traditional Chinese Medicine, in providing mental health care in China; these numbers similarly exclude such practitioners from the Canadian mental health service landscape.

#### Toronto

The Canadian sample consisted of Euro-Canadian psychiatric outpatients from the Depression Clinic of the Mood and Anxiety Program, at the Centre for Addiction and Mental Health in Toronto, Ontario, Canada. Participants were recruited in the Spring and Summer of 2002. Canada has a median age of 40.4 years and a life expectancy of 81.2 years (Central Intelligence Agency, 2009). The city of Toronto serves as the capital of the province of Ontario, located in east-central Canada; the metropolitan area had a population of approximately 4.7 million people in 2001 (Statistics Canada, 2010). Like the Neurosis Clinic in Changsha, the Mood and Anxiety Program is the foremost site for the treatment of mood and anxiety disorders in the region, and is also an active clinical training and research setting.

The Centre for Addiction and Mental Health (CAMH) is one of Canada's foremost institutions for mental health care, with research and teaching mandates through its affiliation with the University of Toronto. Data were collected at a CAMH site that has traditionally emphasized research combined with outpatient care and short-term inpatient care. This particular site is next to the university and close to the central business district, in a mixed-use multi-ethnic neighborhood. The depression clinic is part of the Mood and Anxiety Program, and is the leading site in the region for the treatment of major depressive disorder and dysthymic disorder. Many of the clinic's patients also have co-morbid anxiety, personality, and/or somatoform disorders, although there are also specialized anxiety and personality disorder clinics for patients who do not have a primary depressive diagnosis. Complex cases are often referred to the clinic, sometimes including particularly treatment-resistant patients from other parts of the province of Ontario. Psychopharmacological interventions are delivered by licensed psychiatrists, and cognitive-behavioral and interpersonal interventions are primarily delivered by licensed clinical psychologists.

Patients are referred to the clinic, generally by family physicians who are encouraged to ensure that the patient has a primary diagnosis of depression. Data collection took place as part of a screening procedure for new referrals to the clinic; patients who did not meet criteria for depression, or who showed evidence of psychosis, history of mania, substance use, or neurocognitive problems were referred on to the appropriate clinics at CAMH. Some patients are seen only once, with recommendations sent back to the treating physician. Other patients return to the clinic for one or more follow-up sessions based on clinical need and the mode of intervention chosen. Provided that patients are citizens or permanent residents of Canada, payment for services is covered by government-run health insurance.

#### Site equivalence

The two sites were selected in order to maximize comparability without effacing important cultural variation. Both sites are university-affiliated, are located in the urban cores of provincial capitals of roughly similar size, are well-known and well-regarded in their respective regions, and provide diagnostic and psychological assessment, psychopharmacology, and psychotherapy to patients. However, they also differ in several potentially important respects. The site in Changsha is part of a general hospital, takes primarily self-referrals, and specializes in “neurosis”; the site in Toronto is identified as a specifically psychiatric facility, takes primarily physician-referrals, and specializes in “depression.” This last difference in particular has the potential to greatly influence the kinds of patients that might be included in the two study samples, with pure anxiety and personality disorder cases being seen in Changsha and referred elsewhere in Toronto. Ryder and colleagues (2008) describe the measures taken to lessen this problem. In brief, only patients with at least one of the core symptoms of depression or neurasthenia, across three classification systems, were included in the final study sample.

### Participants

Potential participants at both sites had to meet the following criteria for inclusion in the study: (a) no current evidence of psychosis, mania, or cognitive impairment; (b) aged between 18 and 65; and (c) living within the metropolitan area served by the relevant clinic. Furthermore, as mentioned above, all participants in the final study samples had to endorse at least one core symptom of depression or neurasthenia, across the *DSM-IV*, International Classification of Diseases (ICD-10; World Health Organization, 1992), and Chinese Classification of Mental Disorders (CCMD-2-R; Chinese Medical Association and Nanjing Medical University, 1995) classification systems. The use of these inclusion criteria avoided the limitations of using a single diagnostic category or classification system, which are culturally shaped and therefore can impose culturally biased definitions of disorder and distress. Further details regarding participant recruitment, response rates, and sample characteristics are reported by Ryder and colleagues (2008).

The final Chinese sample was composed of 175 outpatients, including 80 men and 95 women, with a mean age of 31 years (*SD* = 11). Of these, 45 (25.7%) had less than a secondary school education, 68 (38.9%) had completed secondary school, and 62 (35.4%) had completed at least one post-secondary degree. A majority of the participants (81.7%) had received some form of formal treatment in the previous month. Somewhat less than half of the participants (42.3%), first sought formal help for their current problem during that previous month.

The final Euro-Canadian sample was composed of 107 outpatients, including 46 men and 61 women, with a mean age of 36 years (*SD* = 10). Of these, 9 (8.4%) had less than a secondary school education, 31 (29.0%) had completed secondary school, and 67 (62.6%) had completed at least one post-secondary degree^2^. A majority of participants (79.4%) had received some form of formal treatment in the previous month, while a minority of participants (21.5%) had first sought help for their current problem during that month.

### Interview

As described by Ryder and colleagues (2008), all participants completed a clinical interview, which consisted of (a) a Spontaneous Problem Report (SPR) and (b) a Structured Clinical Interview (SCI). The SPR elicited participants' reasons for seeking treatment in an open-ended manner. The first four symptoms or problems reported by each participant were coded into symptom categories, and these categories were grouped into broader symptom classes (i.e., psychological, typical somatic, atypical somatic; see Ryder et al., 2008).

The SCI consisted of a modified version of the Structured Clinical Interview for *DSM–IV*, Axis I, Patient Version, modules for mood disorders (SCID; First et al., 1997). Key modifications were (a) the addition of symptom criteria for depression and neurasthenia from the ICD-10 and the CCMD-2-R; (b) the assessment of all symptoms, regardless of syndrome criteria; and (c) the use of an expanded 0–3 rating scale, to allow for dimensional assessment of symptom severity. Further details regarding the development of the SCI are reported by Ryder and colleagues (2008).

### Analyses

In line with the objectives of their original study, Ryder and colleagues (2008) created psychological and somatic symptom subscales based on the symptoms contained in the SCI, using both a coding scheme and principal axis factor analysis. The current study uses the individual items from these authors' original symptom subscales, to examine cultural variations in the reporting of individual psychological and somatic symptoms. The individual psychological symptoms are suppressed emotions, depressed mood, suicidality, worthlessness/guilt, social avoidance, low self-esteem, loss of interest, and hopelessness. Eight typical somatic symptoms and four reversed (atypical) somatic symptoms were examined in the current study. The typical somatic symptoms were fatigue, psychomotor retardation, insomnia, appetite decrease, weight loss, dizziness, weakness, and pain; the atypical somatic symptoms were weight gain, appetite gain, hypersomnia, and psychomotor agitation.

In order to assess whether particular SCI symptoms (reflected by individual items) behaved differently in the two samples, we conducted analyses using the standardized mean difference technique to assess for DIF among the psychological and typical somatic symptoms. Adjusted means for each individual psychological or typical somatic symptom were calculated by controlling for the appropriate total subscale score; this procedure was conducted for each cultural group separately. For each symptom, the group difference between the adjusted means was then calculated, and expressed as an effect size (Cohen's *d*). Statistically significant effect sizes at the *p* < 0.05 level signaled significant DIF. Items with significant DIF were then removed one-by-one from the total subscale score, beginning with the item displaying the largest effect size. Adjusted means for the remaining items were then re-calculated, along with the group differences on these new adjusted means. This procedure was conducted until none of the remaining items displayed significant DIF.

As there were too few atypical somatic symptoms on the SCI to conduct DIF analyses, individual chi-square tests were conducted to examine group differences in these symptoms. Depending on the results of these analyses, further examination of atypical depression was planned. Specifically, a cross-cultural comparison of the number of outpatients meeting criteria for the AD subtype was planned in the event of consistent group differences in the relevant atypical somatic symptoms.

The SPR results were analyzed as a follow-up to the analysis of the SCI symptoms. Chi-square analyses were conducted to examine group differences in the percentage of participants who spontaneously reported specific somatic and psychological symptoms of interest, in order to complement the DIF and chi-square SCI results. These analyses allowed some examination of the possibility that any DIF findings may be limited to the assessment modality of a structured interview, and offered the opportunity for internal replication of findings regarding specific symptoms.

## Results

### Somatic symptoms

As previously reported by Ryder and colleagues (2008), the Chinese outpatients reported significantly higher levels of typical somatic symptoms, as compared to the Euro-Canadians. The focus of the current analyses was whether or not this pattern of overall symptom reporting would hold true across the individual symptoms. Inspection of the raw means of each symptom indicated that all typical somatic symptoms showed the expected pattern of cross-cultural difference with the exception of pain, which showed essentially no group difference. However, the simple comparison of raw item means is not an appropriate analytical technique, since it is known that the two groups differ in their overall reporting of typical somatic symptoms. In order to understand whether or not particular symptoms are indeed behaving differently than the rest, we need to adjust for overall symptom reporting by controlling for the total subscale score, as described above.

**Figure 1:**
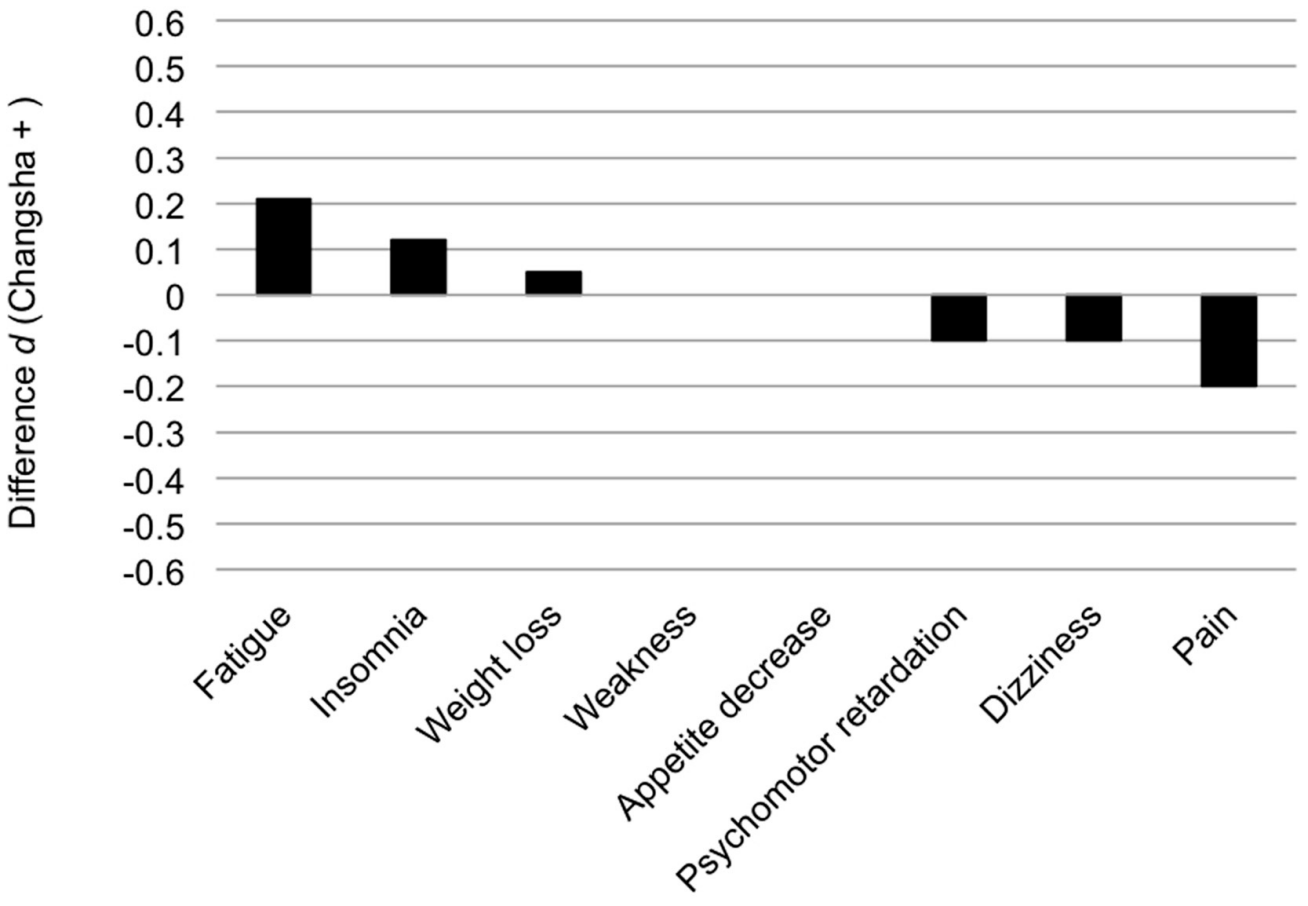
**Group difference in the adjusted means of individual typical somatic symptoms from the Structured Clinical Interview (SCI), expressed as an effect size (Cohen's *d*)**.

None of the typical somatic symptoms showed statistically significant DIF, based on the magnitude of their respective effect sizes (see Figure 1). The effect sizes ranged from *d* = −0.20 to 0.21. In light of the lack of statistical significance, no further DIF analyses were conducted on this set of symptoms.

Chi-square analyses of the atypical somatic symptoms from the SCI showed an unanticipated pattern of results. In contrast to the general pattern of Chinese outpatients endorsing higher levels of somatic symptoms, Euro-Canadian outpatients endorsed three out of the four atypical somatic symptoms significantly more often than did the Chinese. As shown in Table 1, Euro-Canadian outpatients were significantly more likely to report weight gain, appetite gain, and hypersomnia, as compared to the Chinese. Intriguingly, these three atypical somatic symptoms are among the diagnostic symptoms of atypical depression (AD).

**Table 1:** **Atypical somatic symptoms based on the SCI**.

	**Changsha %**	**Toronto %**	***X*^2^**
	**(*n* = 175)**	**(*n* = 107)**	
Weight gain	1.71	15.89	20.24^**^
Appetite gain	1.14	11.21	14.28^**^
Hypersomnia	5.71	17.76	10.44^**^
Psychomotor agitation	22.29	18.69	0.52

*SCI, Structured Clinical Interview*.

** *p ≤ 0.01*.

This pattern of results prompted a follow-up chi-square analysis to examine and compare rates of AD in our two groups. In order to calculate the number of participants in each group who met criteria for AD, the subgroup of participants who met criteria for a *DSM-IV* defined major depressive episode (MDE) had to first be calculated. Eighty-seven Chinese and 69 Euro-Canadians met the *DSM-IV* criteria for an MDE, and were therefore evaluated further to determine whether they met criteria for the AD specifier. Participants qualified for this specifier when two additional criteria were met. First, mood reactivity in response to positive events had to be endorsed. Second, two or more of the following features also had to be endorsed: (1) significant weight gain or increase in appetite; (2) hypersomnia; (3) heavy or leaden feelings in arms or legs; and (4) a long-standing pattern of interpersonal rejection sensitivity, leading to significant social or occupational impairment. Among those participants who met criteria for a *DSM-IV*-defined MDE, Euro-Canadians were significantly more likely than their Chinese counterparts to meet criteria for a diagnosis of the AD subtype (17.4% vs. 4.6%, *X*^2^ = 6.84, *p* < 0.01).

Using the SPR, we then examined the percentage of participants in the two cultural groups who spontaneously reported the somatic symptoms that emerged as potentially important based on the SCI analyses. In the DIF analysis of the SCI, fatigue and pain demonstrated the largest effect sizes, although neither reached conventional levels of significance. Similarly, on the SPR, neither of these symptoms showed a significant group difference. Approximately one quarter of participants reported fatigue in each group (21.14% of Chinese and 28.04% of Euro-Canadians; *X*^2^ = 1.77, *ns*), and just under 10% of participants in each group reported pain (9.71% of Chinese and 9.35% of Euro-Canadians; *X*^2^ = 0.01, *ns*). For the atypical somatic symptoms of increased appetite and hypersomnia, the SPR results paralleled the chi-square analyses of the SCI data, with greater rates of spontaneous reporting among the Euro-Canadians. However, very few participants across both groups spontaneously reported either of these symptoms. Four Euro-Canadians (3.74%) and no Chinese reported increased appetite (*X*^2^ = 6.64, *p* < 0.01), while four Euro-Canadians (3.74%) and one Chinese (0.57%) reported hypersomnia (*X*^2^ = 3.82, *p* < 0.05).

### Psychological symptoms

The analysis of the psychological symptoms largely paralleled that conducted for the somatic symptoms, though without the typical/atypical distinction. As previously reported by Ryder and colleagues (2008), the Chinese outpatients reported significantly fewer psychological symptoms overall, as compared to the Euro-Canadians. Inspection of the raw means of the individual symptoms that constitute the psychological subscale suggested divergent patterns across the symptoms. Although the majority of these symptoms followed the overall pattern of lower levels among the Chinese sample, the magnitude of group difference varied across symptoms, with “hopelessness” showing the largest group difference. Furthermore, two symptoms—depressed mood and suppressed emotions—stood out, as they demonstrated the opposite pattern, with somewhat higher endorsement in the Chinese group. However, as we argued earlier, overall psychological symptom reporting must be taken into account before interpreting these apparent differences across the individual symptoms; hence the need for the DIF analysis.

**Figure 2:**
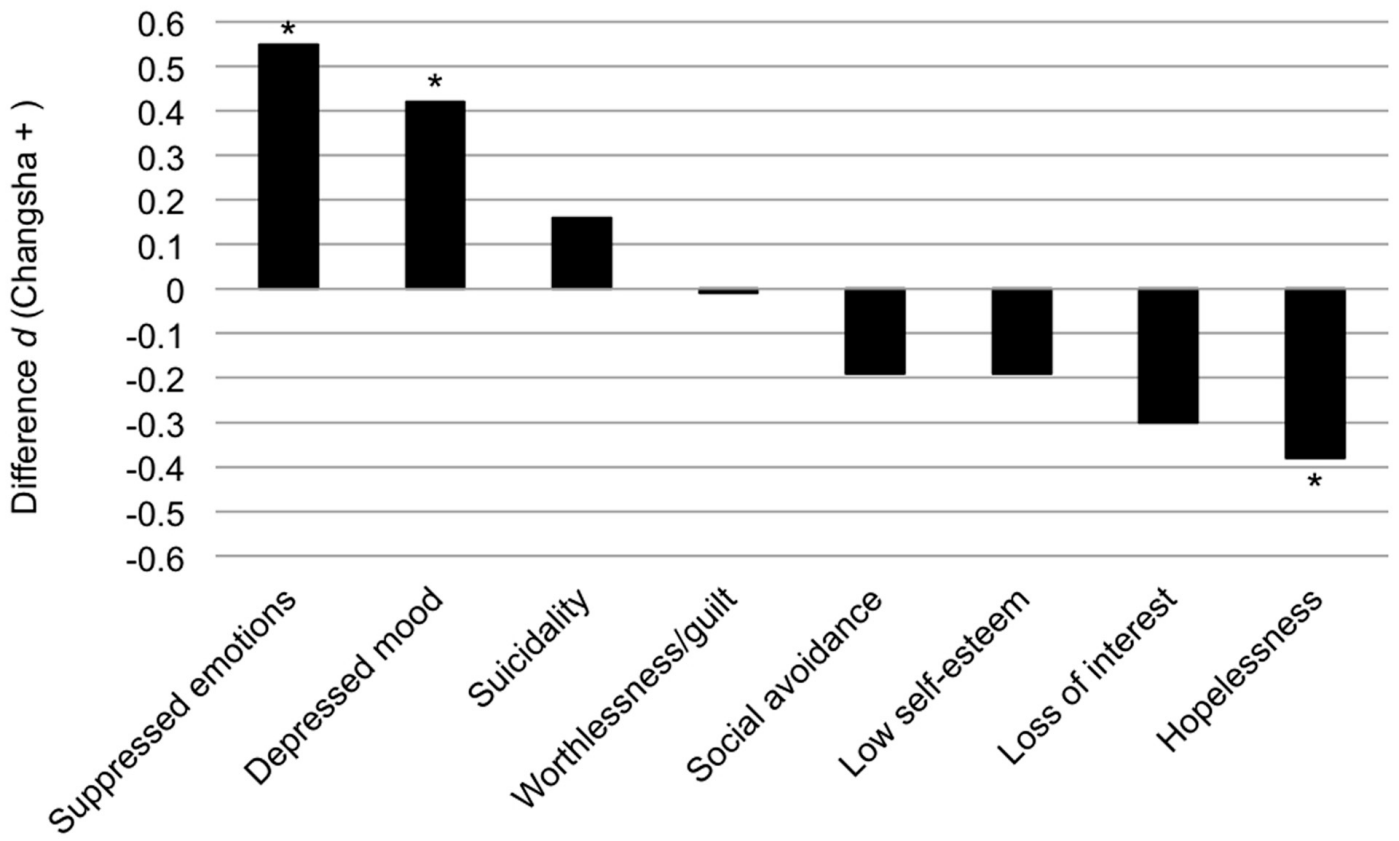
**Group difference in the adjusted means of individual psychological symptoms from the Structured Clinical Interview (SCI), expressed as an effect size (Cohen's *d*).** ^*^p < 0.05.

The DIF analysis revealed that three psychological symptoms—suppressed emotions, depressed mood, and hopelessness—showed statistically significant DIF (see Figure 2). The Chinese reported particularly high levels of suppressed emotions and depressed mood, relative to their overall reporting of psychological symptoms. The group difference on the adjusted means of these two symptoms had large effect sizes (Cohen's *d* of −0.55 and −0.42, respectively). Meanwhile, the Euro-Canadians reported particularly high levels of hopelessness relative to their overall levels of psychological symptom reporting, with the group difference for this item having a large effect size (Cohen's *d* = −0.38). Therefore, controlling for overall psychological symptom reporting, Chinese participants reported higher levels of affective symptoms (suppressed emotions, depressed mood), whereas Euro-Canadian participants seemed to report higher levels of symptoms related to the cognitive elaboration of emotional distress (e.g., hopelessness).

Following the initial DIF analysis, we proceeded to remove symptoms with significant DIF from the subscale one at a time, based on magnitude of effect size. With the removal of suppressed emotions from the total subscale and item-level analysis, depressed mood continued to show significant DIF (Cohen's *d* = −0.50), whereas hopelessness was no longer significant (Cohen's *d* = 0.31). With the removal of both depressed mood and suppressed emotions, there was no further evidence of significant DIF for any of the remaining items.

In order to supplement these findings, we examined the extent to which the Chinese and Euro-Canadian outpatients spontaneously reported the three symptoms of interest from the DIF analysis of the SCI symptoms (depressed mood, suppressed emotions, and hopelessness). We were particularly interested in the extent to which Chinese outpatients spontaneously reported depressed mood, in light of the discrepancy between the traditional notion that people of Chinese heritage are reluctant to report emotional distress and the DIF finding of relatively high levels of depressed mood being reported among the Chinese outpatients. Chi-square analyses revealed no significant group difference in spontaneously reported depressed mood, with 56% of Chinese and 48.6% of Euro-Canadians reporting this symptom on the SPR (*X*^2^ = 1.46, *ns*). This result is quite surprising given the central role that denial of depressed mood plays in descriptions of Chinese symptom presentations; however, this result is consistent with the DIF findings in that depressed mood did not follow the overall pattern of lower levels of psychological symptom reporting in the Chinese group.

Although the spontaneous reporting of suppressed emotions was very low in both groups, a greater percentage of Chinese outpatients reported this symptom as compared to the Euro-Canadians (5.1% vs. 0.9%), in line with the DIF results; however, the chi-square analysis was not statistically significant (*X*^2^ = 3.44, *ns*). Finally, the spontaneous reporting of hopelessness also fit with the DIF results. A significantly greater percentage of Euro-Canadians spontaneously reported this symptom than the Chinese (8.4% vs. 1.1%; *X*^2^ = 9.36, *p* < 0.01). These results strengthen the DIF findings by suggesting that Chinese outpatients report comparable or higher levels of affect-related symptoms as compared to Euro-Canadians not only when asked structured interview questions, but also when spontaneously reporting their symptoms.

## Discussion

“Somatization” and “psychologization,” in recent understandings, describe the tendency to emphasize somatic or psychological symptoms when presenting psychopathology. The assumption here is that there is something in general about somatic symptoms or psychological symptoms that lead them to be emphasized, as a set. Our aim was to determine whether this assumption holds true, or whether a narrower subset of symptoms are responsible for observed cultural variation in comparisons of Chinese and “Western” samples. Is the traditional idea of neurasthenia in China reflected in particularly high rates of fatigue and insomnia in Chinese vs. “Western” depressed psychiatric outpatients? Are Chinese patients particularly unlikely to endorse depressed mood? Are the reversed, atypical symptoms simply additional examples of somatic symptoms or do they show their own pattern of cultural variation? We sought to examine such questions.

DIF analysis of the typical somatic symptoms found no specific symptoms that showed a significantly divergent pattern from the overall subscale. We observed striking cultural variation for the reversed somatic symptoms, however, such that Euro-Canadian participants reported significantly higher levels of weight and appetite gain and hypersomnia. As these symptoms are part of the atypical depression subtype described in *DSM-IV*, we compared rates of this subtype in the two samples and found that atypical depression was considerably more common among the Euro-Canadians. Higher rates of reversed somatic symptoms and of atypical depression in Euro-Canadian outpatients, compared with Chinese outpatients, suggest that there may be forms of somatization that are more common in “Western” contexts. Given the association between atypical depression and somatization discussed earlier, these results underscore the need for caution when claiming that particular cultural groups “somatize” in general.

One possible explanation for this unexpected finding is that atypical depression reflects a particular personality style that is more common in some cultural milieus than in others. Atypical depression is unusual among Axis I diagnostic categories in that one of its symptoms is a personality trait: a longstanding pattern of interpersonal rejection sensitivity, which may be a better indicator for detecting atypical depression than mood reactivity, its current primary symptom (Parker, 2007; Thase, 2009). There is evidence that although there are higher rates of shyness and other normal interpersonal concerns in Chinese cultural contexts, clinically significant symptoms of interpersonal dysfunction are less common (Zhou et al., 2011). Given that this trait is associated with a pattern of reversed somatic symptoms, at least in “Western” samples (Parker et al., 2002; Angst et al., 2006), cultural variation in personality may drive this particular form of somatization. Another possibility, however, is that these findings are a consequence of using a clinical sample—it may be that reversed somatic symptoms and the atypical syndrome are less likely to be identified as problems requiring mental health intervention in the Chinese cultural context. Future research will be required to determine whether the source of this variation lies in symptom presentation or patterns of help-seeking. Such work would benefit, moreover, from direct assessment of participants' physical health, as our interpretations of somatic symptom differences—whether typical or atypical—remain limited by a lack of such information in the current study (see Kohrt et al., 2005).

Turning to psychologization, Chinese participants reported significantly higher levels of suppressed emotions and depressed mood after controlling for the overall tendency of Euro-Canadian participants to report more psychological symptoms. Once these symptoms were removed from the psychological symptom set, there was no further evidence of DIF. The “suppressed emotions” item merits a brief comment, as the label may be misleading. This symptom was assessed with the interview question: “have you been feeling like you no longer have emotional responses to events that would normally affect you emotionally?” Therefore, although endorsement of this item signifies a lack of emotional responding, it also reflects attention to one's emotional life and a willingness to report shifts in one's emotional experiences. Combined with the pattern of endorsement of depressed mood, these results call into question the common assumption that Chinese patients are unwilling to discuss emotional distress or emotion-related symptomatology.

Indeed, although our research generally supports long-established cultural variations in symptom presentation, these more detailed findings show that the picture has changed in some important ways. In Kleinman's (1982) original study, semi-structured interview techniques were used to carefully probe the symptom experiences of neurasthenia patients, leading to a re-diagnosis of most cases with some form of depression. Nonetheless, the spontaneously reported presenting complaints and unstructured accounts of most of these patients emphasized somatic symptoms with a minimal role for depressed mood, reflecting a neurasthenia script held by patients and Chinese mental health professionals at the time. We conducted our research at the same facility, a quarter-century or so later, where the neurasthenia diagnosis is now rarely used. Although the patients still emphasized somatic symptoms relative to a Euro-Canadian comparison group, their symptom presentation tended not to follow the older neurasthenia script. Instead, the patients' spontaneous reports reveal a script that includes depressed mood, described with words that are at least roughly equivalent to “depressed” and “sad.” Emphasis on the somatic is not minimization or denial of depressed mood. Available cultural scripts for the presentation of emotional distress and social suffering appear to have changed markedly over a generation (Ryder et al., 2012).

The psychological symptom emphasis observed in Euro-Canadian relative to Chinese psychiatric outpatients relates more to the cognitive elaboration of emotional distress rather than to variation in whether such distress is reported in the first place: symptoms such as hopelessness are central to this pattern of cultural variation, whereas depressed mood is not. This distinction may be attributable to the relative importance placed on emotional experience and expression in the two cultural contexts. Cultural scripts shape the ways in which people attend and react to particular experiences marked as important in some way. In some cases pathological loops can form, where attention to a particular symptom can accentuate its severity and give rise to related symptoms (Ryder and Chentsova-Dutton, 2012). Symptoms such as hopelessness may be particularly common in cultural contexts that foster cognitive and behavioral elaboration of negative emotional states.

Again, however, the sample composition places limits on interpretation. This study was conducted in psychiatric clinical samples where participants were already identified as suffering from some kind of mental health problem. Denial or minimization of depressed mood may be more common in, for example, general practice or internal medicine contexts where a subset of patients may be depressed, but with the depression unrecognized due to a paucity of psychological symptoms. Chinese patients in rural areas, with lower exposure to modern technologies and “Western” ideas about psychopathology, may also exhibit a stronger tendency to not endorse depressed mood. Studies conducted in different contexts, with different kinds of samples, will be necessary to address these possibilities.

What this research does suggest is that there is value in considering affective and cognitive psychologization as somewhat different phenomena, at least when considering Chinese vs. “Western” comparisons. On the one hand, these two symptom sets are highly correlated, loading on a single factor, and their DIF is not pronounced enough to preclude cultural comparison using a single composite scale (Ryder et al., 2008). Nonetheless, there may be sufficient variation in the cultural meaning attributed to emotional and cognitive symptoms that research in this area would be well-served by instruments that can effectively separate them. Indeed, recognizing this affective vs. cognitive distinction is in line with other recent work in cultural psychology (e.g., Ma-Kellams et al., 2011). There is already evidence that psychologization is a consistently stronger effect than somatization when comparing Chinese and “Western” samples (Parker et al., 2001a; Ryder et al., 2008), and it is possible that this effect was actually underestimated due to the inclusion of affective symptoms such as depressed mood. Separate consideration of these symptom classes would allow future researchers to begin the process of unpacking the underlying cultural mechanisms that explain variations specific to cognitive symptoms.

Indeed, a notable weakness of the current analyses is the lack of methodological unpacking of possible cultural explanations. We are committed to a vision of cultural-clinical psychology in which potential explanations for observed variations are tested directly rather than offered on a *post-hoc* basis (Ryder and Dere, 2010; Ryder et al., 2011). Indeed, the original study boasted exactly this approach, showing that cultural variation in somatization could be partially explained by externally-oriented thinking (see Ryder et al., 2008). Recent studies have further shown that variation in externally-oriented thinking can be explained in part by differences in cultural values (Dere et al., 2012, 2013). Unfortunately, this approach is only possible when one begins with clear explanatory hypotheses. The current analyses were not specifically designed to unpack cultural variation in atypical depression or cognitive psychologization. The next step for future research is to directly test possible explanations for the results that have emerged here. We therefore conclude with some specific proposals as to how this might be done, with the aim of promoting future advances in cultural-clinical psychology as applied to depression.

Studies designed to help explain and build upon the current pattern of results should be informed by other recent cultural psychology studies of depression. Work by Chentsova-Dutton and colleagues (Chentsova-Dutton et al., 2007, 2010) on the cultural norm hypothesis is particularly relevant. This hypothesis posits that depression impairs people's ability to successfully enact cultural emotion norms and, therefore, that those with depression will display patterns of emotional responding that diverge from their cultural ideals. Support for this hypothesis has been found in two samples of depressed and non-depressed Euro-Americans and Asian-Americans, using an experimental research design with emotion eliciting film clips. Depressed Euro-Americans showed dampened emotional responding compared to non-depressed controls, countering cultural norms emphasizing open emotional expression. Depressed Asian-Americans, by contrast, showed similar or greater emotional responding compared to non-depressed controls, countering cultural norms of emotional moderation. This work suggests that studying emotional reactivity in depression can help us to better understand normative cultural expectations regarding emotion, thereby illustrating an important objective of cultural-clinical psychology research.

Although relevant, Chentsova-Dutton and colleagues' (2007, 2010) conclusions do not clearly align with the current findings. We found that Chinese outpatients reported higher levels of suppressed emotions, which seems to be in line with, rather than counter to, local emotion norms. This seeming challenge to the cultural norm hypothesis might be attributable to several possible explanations, including substantial differences in study methodology and mode of reporting, as well as sample differences. Future work seeking to reconcile these lines of research, perhaps by integrating an experimental design with the examination of specific symptom reporting, would be valuable to the cultural study of depression.

Although a promising line of recent research, work on the cultural norm hypothesis currently also lacks the inclusion of “unpacking” variables. From a theoretical perspective, relevant explanatory variables should include culturally-based values regarding emotional expression. The role of such values in mediating emotional responding in the context of depression has not yet been examined. Here, the work of Mauss and colleagues (Mauss and Butler, 2010; Mauss et al., 2010) on emotion control values (ECVs) merits attention. ECVs refer to the extent to which one believes that people should generally control their emotions, and tend to be more strongly endorsed by Asian- as compared to Euro-Americans. Such values may also provide important explanatory power regarding the current findings. In particular, the assessment of emotion related values might help to clarify cultural variations in the reporting of affective vs. cognitive psychological symptoms. Future work would therefore benefit from the integration of these recent lines of research, with the aim of moving beyond demonstration of cultural differences in depressive symptom reporting or emotional responding, and toward explanatory models of such differences.

Finally, in addition to the need for integration with other recent work in this area, the current findings also point toward a number of new lines of inquiry. In particular, studies seeking to explain the divergent pattern of results between typical and atypical somatic symptom reporting are likely to offer novel insights into our understanding of cultural variations in somatization. Drawing on the recent discussion of Chinese somatization by Ryder and Chentsova-Dutton (2012), potential explanatory variables to consider include perceived stigma, access and barriers to services, perceived thresholds of symptom severity, and measures that tap into attentional processes. Studies examining the role of attentional processes could build on previous findings regarding externally-oriented thinking as mentioned above (Dere et al., 2012, 2013; Ryder et al., 2008), as well as relevant work on cultural differences in somatic- vs. affective-focused attention (e.g., Chen et al., 2003; Dzokoto, 2010). Such work has the potential to make a significant contribution not only to the cultural literature on somatization and depressive symptom presentation, but also to our understanding of cultural scripts regarding emotion and the body at times of profound distress.

### Conflict of interest statement

The authors declare that the research was conducted in the absence of any commercial or financial relationships that could be construed as a potential conflict of interest.

## Footnotes

^1^Both the clinic and affiliated university have had name changes since the time of the original data collection, and this setting is now the Psychology Clinic of the Medical Psychological Research Institute at the Second Xiangya Hospital, Central South University.

^2^As reported by Ryder and colleagues (2008), the Euro-Canadian group had a significantly higher education level as compared to the Chinese group. Ryder and colleagues (2008) included education as a covariate in follow-up analyses examining the group differences in somatic and psychological symptom reporting, and found no change in the pattern of results with the SCI subscales. At the suggestion of a reviewer, we further explored the potential role of education level in our findings by conducting correlational analyses (Spearman's rho) between education and the somatic and psychological SCI subscales, separately in the two cultural groups. The only significant correlation was between education and somatic symptom reporting in the Chinese group (*r* = −0.21, *p* < 0.01). Combined with the findings of Ryder and colleagues (2008), these results suggests that the difference in education level between the two groups does not represent a significant confound.

## References

[B1] Akiskal H. S. Benazzi F. (2005). Atypical depression: a variant of bipolar II or a bridge between unipolar and bipolar II? J. Affect. Disord. 84, 209–217 10.1016/j.jad.2004.05.004 15708418

[B2] American Psychiatric Association. (1980). Diagnostic and Statistical Manual of Mental Disorders, 3rd Edn. Washington, DC: Author

[B3] American Psychiatric Association. (1994). Diagnostic and Statistical Manual of Mental Disorders, 4th Edn. Washington, DC: Author

[B4] Angst J. Gamma A. Benazzi F. Silverstein B. Ajdacic-Gross V. Eich D. (2006). Atypical depressive syndromes in varying definitions. Eur. Arch. Psychiatry Clin. Neurosci. 256, 44–54 10.1007/s00406-005-0600-z 16041559

[B5] Barsky A. J. (1992). Amplification, somatization, and the somatoform disorders. Psychosomatics 33, 28–34 10.1016/S0033-3182(92)72018-0 1539100

[B6] Benazzi F. (1999). Atypical depression in private practice depressed outpatients: a 203-case study. Compr. Psychiatry 40, 80–83 10.1016/S0010-440X(99)90081-0 9924882

[B7] Central Intelligence Agency. (2009). The World Factbook 2009. Washington, DC: Author

[B8] Chen H. Guarnaccia P. J. Chung H. (2003). Self-attention as a mediator of cultural influences on depression. Int. J. Soc. Psychiatry 49, 192–203 10.1177/00207640030493005 14626362

[B9] Chentsova-Dutton Y. Chu J. P. Tsai J. L. Rottenberg J. Gross J. J. Gotlib I. H. (2007). Depression and emotional reactivity: variation among Asian Americans of East Asian descent and European Americans. J. Abnorm. Psychol. 116, 776–785 10.1037/0021-843X.116.4.776 18020723

[B10] Chentsova-Dutton Y. Tsai J. L. Gotlib I. H. (2010). Further evidence for the cultural norm hypothesis: positive emotion in depressed and control European American and Asian American women. Cult. Divers. Ethnic Minor. Psychol. 16, 284–295 10.1037/a0017562 20438167 PMC2864927

[B11] Chinese Medical Association Nanjing Medical University. (1995). Chinese Classification of Mental Disorders, 2nd Edn., revised (CCMD-2-R). Nanjing: Dong Nan University Press [in Chinese]

[B12] Dere J. Falk C. F. Ryder A. G. (2012). Unpacking cultural differences in alexithymia: the role of cultural values among Euro-Canadian and Chinese-Canadian students. J. Cross. Cult. Psychol. 43, 1297–1312 10.1177/0022022111430254

[B13] Dere J. Tang Q. Zhu X. Cai L. Yao S. Ryder A. G. (2013). The cultural shaping of alexithymia: values and externally oriented thinking in a Chinese clinical sample. Compr. Psychiatry 54, 362–368 10.1016/j.comppsych.2012.10.013 23261082

[B14] Dzokoto V. (2010). Different ways of feeling: emotion and somatic awareness in Ghanaians and Euro-Americans. J. Soc. Evol. Cult. Psychol. 4, 68–78

[B15] First M. B. Spitzer R. L. Gibbon M. Williams J. B. W. (1997). Structured Clinical Interview for DSM-IV Axis I Disorders, Research Version, Patient Edition (SCID-I/P). New York, NY: Biometrics Research, New York State Psychiatric Institute

[B16] Hambleton R. K. Swaminathan H. Rogers H. J. (1991). Fundamentals of Item Response Theory. Thousand Oaks, CA: Sage Publications, Inc

[B17] Horwath E. Johnson J. Weissman M. M. Hornig C. D. (1992). The validity of major depression with atypical features based on a community study. J. Affect. Disord. 26, 117–125 10.1016/0165-0327(92)90043-6 1447429

[B19] Kirmayer L. J. Robbins J. M. (1991). Three forms of somatization in primary care: prevalence, co-occurrence, and socio-demographic characteristics. J. Nerv. Ment. Dis. 179, 647–655 10.1097/00005053-199111000-00001 1940887

[B20] Kirmayer L. J. Young A. (1998). Culture and somatization: clinical, epidemiological, and ethnographic perspectives. Psychosom. Med. 60, 420–430 9710287 10.1097/00006842-199807000-00006

[B21] Kleinman A. (1982). Neurasthenia and depression: a study of somatization and culture in china. Cult. Med. Psychiatry 6, 117–190 10.1007/BF00051427 7116909

[B22] Kleinman A. M. (1977). Depression, somatization and the new cross-cultural psychiatry. Soc. Sci. Med. 11, 3–10 10.1016/0037-7856(77)90138-X 887955

[B23] Kohrt B. A. Kunz R. D. Baldwin J. L. Koirala N. R. Sharma V. D. Nepal M. K. (2005). ‘Somatization’ and ‘comorbidity’: a study of jhum-jhum and depression in rural Nepal. Ethos 33, 125–147 10.1525/eth.2005.33.1.125

[B24] Lee S. Ng K. L. Tsang A. (2009). Prevalence and correlates of depression with atypical symptoms in Hong Kong. Aust. N. Z. J. Psychiatry 43, 1147–1154 10.3109/00048670903279895 20001414

[B26] Liu J. Ma H. He Y.-L. Xie B. Xu Y.-F. Tang H.-Y. (2011). Mental health system in China: history, recent service reform and future challenges. World Psychiatry 10, 210–216 10.1002/j.2051-5545.2011.tb00059.x 21991281 PMC3188776

[B27] Ma-Kellams C. Spencer-Rodgers J. Peng K. (2011). I am against us? unpacking cultural differences in in-group favoritism via dialecticism. Pers. Soc. Psychol. Bull. 37, 15–27 10.1177/0146167210388193 21084525

[B28] Mauss I. B. Butler E. A. (2010). Cultural context moderates the relationship between emotion control values and cardiovascular challenge versus threat responses. Biol. Psychol. 84, 521–530 10.1016/j.biopsycho.2009.09.010 19786064 PMC2950892

[B29] Mauss I. B. Butler E. A. Roberts N. A. Chu A. (2010). Emotion control values and responding to an anger provocation in Asian-American and European-American individuals. Cogn. Emot. 24, 1026–1043 10.1080/02699930903122273 21116444 PMC2992431

[B30] National Bureau of Statistics of China. (2002). Tabulation on the 2000 Population Census of the People's Republic of China. China Statistics Press. Available online at: http://tongji.cnki.net/overseas/engnavi/YearBook.aspx?id=N2005121054&floor=1 (Accessed December 15, 2012).

[B31] Parker G. B. (2007). Atypical depression: a valid subtype? J. Clin. Psychiatry 68(Suppl. 3), 18–22 17348763

[B32] Parker G. Cheah Y.-C. Roy K. (2001a). Do the Chinese somatize depression? A cross-cultural study. Soc. Psychiatry Psychiatr. Epidemiol. 36, 287–293 10.1007/s001270170046 11583458

[B33] Parker G. Gladstone G. Tsee Chee K. (2001b). Depression in the planet's largest ethnic group: the Chinese. Am. J. Psychiatry 158, 857–864 10.1176/appi.ajp.158.6.857 11384889

[B34] Parker G. Roy K. Mitchell P. Wilhelm K. Malhi G. Hadzi-Pavlovic D. (2002). Atypical depression: a reappraisal. Am. J. Psychiatry 159, 1480–1481 10.1176/appi.ajp.159.9.1480 12202264

[B36] Robertson H. A. Lam R. W. Stewart J. N. Yatham E. M. (1996). Atypical depressive symptoms and clusters in unipolar and bipolar depression. Acta Psychiatr. Scand. 94, 421–427 10.1111/j.1600-0447.1996.tb09884.x 9020993

[B37] Ryder A. G. Ban L. M. Chentsova-Dutton Y. E. (2011). Towards a cultural–clinical psychology. Soc. Pers. Psychol. Compass 5, 960–975 10.1111/j.1751-9004.2011.00404.x

[B38] Ryder A. G. Chentsova-Dutton Y. (2012). Depression in cultural context: “Chinese somatization,” revisited. Psychiatr. Clin. North Am. 35, 15–36 10.1016/j.psc.2011.11.006 22370488

[B39] Ryder A. G. Dere J. (2010). Canadian diversity and clinical psychology: defining and transcending ‘cultural competence’. Coll. Alberta Psychol. Monit. 35, 6–13

[B40] Ryder A. G. Sun J. Zhu X. Yao S. Chentsova-Dutton Y. (2012). Depression in China: integrating developmental psychopathology and cultural-clinical psychology. J. Clin. Child Adolesc. Psychol. 41, 682–694 10.1080/15374416.2012.710163 22900498

[B41] Ryder A. G. Yang J. Zhu X. Yao S. Yi J. Heine S. J. (2008). The cultural shaping of depression: somatic symptoms in china, psychological symptoms in North America? J. Abnorm. Psychol. 117, 300–313 10.1037/0021-843X.117.2.300 18489206

[B42] Seemüller F. Riedel M. Wickelmaier F. Adli M. Mundt C. Marneros A. (2008). Atypical symptoms in hospitalized patients with major depressive episode: frequency, clinical characteristics, and internal validity. J. Affect. Disord. 108, 271–278 10.1016/j.jad.2007.10.025 18164767

[B43] Simon G. E. VonKorff M. Piccinelli M. Fullerton C. Ormel J. (1999). An international study of the relation between somatic symptoms and depression. New Engl. J. Med. 341, 1329–1335 10.1056/NEJM199910283411801 10536124

[B44] Statistics Canada. (2010). Population and Dwelling Counts, for Urban Areas 2006 and 2001 Censuses - 100% Data. Available online at: http://www12.statcan.gc.ca/census-recensement/2006/dppd/hlt/97550/Index.cfm?TPL=P1C&Page=RETR&LANG=Eng&T=801&SR=1&S=1&O=A&RPP=9999&PR=0&CMA=0 (Accessed December 16, 2012).

[B45] Sullivan P. F. Kessler R. C. Kendler K. S. (1998). Latent class analysis of lifetime depressive symptoms in the national comorbidity survey. Am. J. Psychiatry 155, 1398–1406 9766772 10.1176/ajp.155.10.1398

[B46] Thase M. E. (2009). Atypical depression: useful concept, but its time to revise the DSM-IV criteria. Neuropsychopharmacology 34, 2633–2641 10.1038/npp.2009.100 19741592

[B47] Thuile J. Even C. Musa C. Friedman S. Rouillon F. (2009). Clinical correlates of atypical depression and validation of the French version of the scale for atypical symptoms (SAS). J. Affect. Disord. 118, 113–117 10.1016/j.jad.2009.02.005 19272652

[B48] Tsai J. L. Simeonova D. I. Watanabe J. T. (2004). Somatic and social: Chinese Americans talk about emotion. Pers. Soc. Psychol. Bull. 30, 1226–1238 10.1177/0146167204264014 15359024

[B49] World Health Organization. (1992). International Statistical Classification of Diseases and related health Problems (ICD-10), Tenth Revision. Geneva: Author

[B50] World Health Organization. (2011). Ment. Health Atlas 2011. Geneva: Author

[B52] Zhou X. Dere J. Zhu X. Yao S. Chentsova-Dutton Y. Ryder A. G. (2011). Anxiety symptom presentations in Han Chinese and Euro-Canadian outpatients: is distress always somatized in China? J. Affect. Disorders 135, 111–114 10.1016/j.jad.2011.06.049 21794924

[B54] Zumbo B. D. (2007). Three generations of DIF analyses: considering where it has been, where it is now, and where it is going. Lang. Assess. Q. 4, 223–233 10.1080/15434300701375832

